# Seroprevalence and Risk Factors of *Chlamydia* Infection in Cattle in Shanxi Province, North China

**DOI:** 10.3390/ani13020252

**Published:** 2023-01-10

**Authors:** Xiao-Jing Wu, Jin Gao, Qian Zhang, Chen-Xu Li, Wen-Bin Zheng, Qing Liu, Xing-Quan Zhu, Yu-Ping Lei, Wen-Wei Gao

**Affiliations:** 1 College of Veterinary Medicine, Shanxi Agricultural University, Jinzhong 030801, China; 2 Key Laboratory of Veterinary Public Health of Higher Education of Yunnan Province, College of Veterinary Medicine, Yunnan Agricultural University, Kunming 650201, China; 3 Veterinary Laboratory, Shanxi Provincial Animal Disease Prevention and Control Center, Taiyuan 030008, China

**Keywords:** *Chlamydia*, *Chlamydia abortus*, cattle, seroprevalence, Shanxi Province

## Abstract

**Simple Summary:**

*Chlamydia*, an important zoonotic pathogen, poses a serious threat to public health and the development of animal husbandry. Shanxi Province, famous for its Loess Plateau terrain, has been taking cattle and sheep husbandry as an important direction of development, which means that it is necessary to prioritize the detection of pathogens in cattle and sheep. In this study, based on the location and management pattern, the seroprevalence of *Chlamydia* and *C. abortus* infection in cattle in Shanxi Province was examined by indirect hemagglutination assay (IHA) and indirect enzyme-linked immunosorbent assay (ELISA), respectively. The results revealed a high (52.29%) overall seroprevalence of *Chlamydia* in cattle in Shanxi Province. Location was closely related to the prevalence of *Chlamydia* and *C. abortus*, and different management patterns also had a certain impact on the prevalence of *Chlamydia*. This study provided valuable baseline information for the prevention and control of *Chlamydia* in cattle in Shanxi Province.

**Abstract:**

The information on *Chlamydia* infection in cattle is limited in Shanxi Province, north China. This study aimed to investigate the seroprevalence and risk factors of *Chlamydia* and *Chlamydia abortus* infection in cattle in Shanxi Province. In November 2020, a large-scale investigation of *Chlamydia* seroprevalence was conducted on 981 cattle serum samples collected from 40 cattle farms in 11 cities of Shanxi Province. The seroprevalence of *Chlamydia* and *C. abortus* was examined by indirect hemagglutination assay (IHA) and enzyme-linked immunosorbent assay (ELISA), respectively. The seroprevalence of *Chlamydia* and *C. abortus* was 52.29% (513/981) and 2.96% (29/981), respectively, in cattle in Shanxi Province. Location was identified as a risk factor for *Chlamydia* and *C. abortus* infection (*p* < 0.05). Under different management patterns, the seroprevalence of *Chlamydia* and *C. abortus* in large-scale animal farming companies was higher than that in household animal farms and animal farming cooperatives, and only the seroprevalence of *Chlamydia* was significantly different in different management patterns (*p* < 0.01). The results showed that there was higher seroprevalence of *Chlamydia* in cattle in Shanxi Province, while *C. abortus* was not the dominant species. This study provided baseline information on *Chlamydia* infection in cattle in Shanxi Province, which constitutes valuable data for monitoring livestock health and preventing potential zoonoses.

## 1. Introduction

*Chlamydia* organisms are obligate intracellular pathogens distributed globally, known to cause various forms of diseases in humans and other vertebrate animals [1]. To date, more than 15 species of *Chlamydia* have been identified [2], of which *Chlamydia pecorum*, *Chlamydia abortus* and *Chlamydia psittaci* have been reported in cattle [3]. *Chlamydia* infection in cattle can cause acute but rare diseases, such as sporadic bovine encephalomyelitis, abortion, enteritis, and pneumonia [3,4]. In addition to the infrequent acute infections, more subclinical infections have been reported in cattle [5,6]. Nevertheless, subclinical infections also have a substantial impact on livestock productivity, including reduced weight gain and infertility [5].

Abortion causes devastating economic losses to the cattle industry, with a total loss of up to GBP 630 per aborted cow [7]. For Iranian Holstein cows, abortion had potential impacts on milk production, fat and protein content in milk of normal calving cows [8]. *C. abortus* is an important pathogen associated with abortion in cattle [9]. In addition, *C. abortus* is a zoonotic species of public health importance, which is responsible for abortion and reproductive problems in humans and other animals [10,11,12].

The mountainous area accounts for 80.1% of the total area in Shanxi Province, which is a typical mountain plateau covered by loess. Therefore, the development of animal husbandry based on cattle and sheep has always been of economic importance to Shanxi Province. At present, the management pattern of cattle farming in Shanxi Province is mostly household animal farming, and the development of large-scale animal farming companies is the future development trend of cattle industry in Shanxi Province. Therefore, understanding the potential risk factors of *Chlamydia* infection is conducive to the transformation of the cattle industry in Shanxi Province.

Serological surveillance of these pathogens in cattle is of great importance. To date, information on the prevalence of *Chlamydia* and *C. abortus* in cattle Shanxi Province is very limited. Therefore, the purpose of this study was to investigate the seroprevalence and risk factors of *Chlamydia* and *C. abortus* infection in cattle in Shanxi Province, and the study was expected to provide a reference for the prevention and control of these pathogens and the development of the cattle industry.

## 2. Materials and Methods

### 2.1. Ethics Approval

The experimental procedures of the study were reviewed and approved by the Animal Research Ethics Committee of Shanxi Agricultural University (Approval No. 2019IACUCSXAU002A01). The animals were handled in accordance with good animal practice as defined by the relevant Animal Ethics Procedures and Guidelines of the People’s Republic of China.

### 2.2. Study Sites

**Figure 1:**
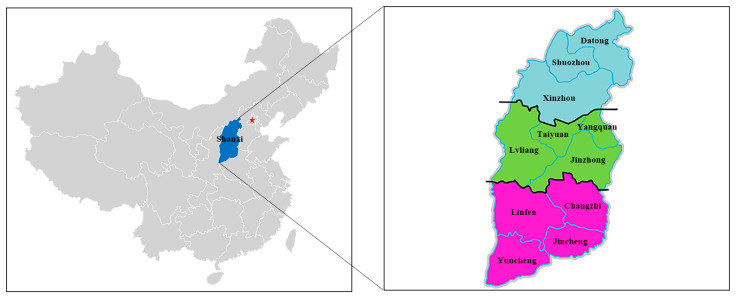
Map showing the geographical locations of 11 administrative cities in Shanxi Province, north China, where cattle blood samples were collected. Sky blue represents north Shanxi Province; grass green represents central Shanxi Province; magenta represents south Shanxi Province. Red pentagram represents Beijing city, the capital of China.

In this study, serum samples were collected from 11 administrative cities in Shanxi Province, North China. Shanxi Province (34°36′–40°44′ N, 110°15′–114°32′ E), located at the east of the Loess Plateau, has a temperate continental monsoon climate with an average annual temperature of 3–14 °C (Figure 1). In the past ten years, the resident population of Shanxi Province has gradually decreased, and more and more of the rural population choose to live in cities. The number of people working in primary industries (agriculture, forestry, husbandry or fishing) has also dropped sharply (Table 1) (http://tjj.shanxi.gov.cn/tjsj/tjnj/nj2021/zk/indexch.htm, accessed on 18 December 2022).

### 2.3. Sample Collection

In this study, 981 serum samples of cattle were collected from 40 cattle farms in 11 cities in Shanxi Province in November 2020; of which 267, 360, and 354 samples were collected from northern (Datong (n = 88), Shuozhou (n = 89) and Xinzhou (n = 90)), central (Taiyuan (n = 90), Lvliang (n = 90), Jinzhong (n = 90) and Yangquan (n = 90)), and southern Shanxi Province (Changzhi (n = 90), Jincheng (n = 90), Linfen (n = 84) and Yuncheng (n = 90)), respectively (Figure 1 and Table 2). At least 3 cattle farms were selected for sampling in each city. The 40 cattle farms included 26 household animal farms (n = 567, stock < 200), 5 animal farming cooperatives (n = 100, stock consists of multiple domestic animal cattle farms) and 9 large-scale animal farming companies (n = 314, stock > 900). After standing at room temperature for 2 h, the samples were centrifuged at 3000 *g* for 10 min to collect the serum and sera were stored at −20 °C for further analysis.

### 2.4. Serological Tests

Antibodies to *Chlamydia* were tested by using indirect hemagglutination assay (IHA) with a commercially available Kit (Lanzhou Veterinary Research Institute, Chinese Academy of Agricultural Sciences, Lanzhou, China), and serum samples were considered as positive for *Chlamydia* if layers of agglutinated erythrocytes were formed in wells at dilutions of 1:16 or greater. Results returning values between 1:4 and 1:16 were considered “suspicious samples” and were tested again. The high sensitivity (100%) and specificity (95%) of this IHA kit guarantee the authenticity of the experimental data [13].

Antibodies to *C. abortus* were examined by using the ID Screen^®^ *C. abortus* Indirect Multi-species ELISA kit (Innovative Diagnostics, France). According to the manufacturer’s specifications, the sample was tested and OD value was measured at 450 nm. The ratio was calculated according to the following formula:

The value = (OD sample/OD positive control) × 100

Samples with values greater than 60% were considered as positive; values equal to or less than 50% as negative; values between 50% and 60% were considered suspicious and were tested again.

A second test for *Chlamydia* (IHA) and *C. abortus* (ELISA) was considered positive if the results remained suspicious.

### 2.5. Statistical Analyses

The seroprevalence and the factors (location and management pattern) for *Chlamydia* and *C. abortus* infection in cattle in Shanxi Province were evaluated by using the SPSS 26 version software (IBM, Armonk, NY, USA). *p* < 0.05 was considered statistically significant. The odds ratios and the 95% confidence interval (CI) of each factor were analyzed in this study.

## 3. Results 

**Figure 2:**
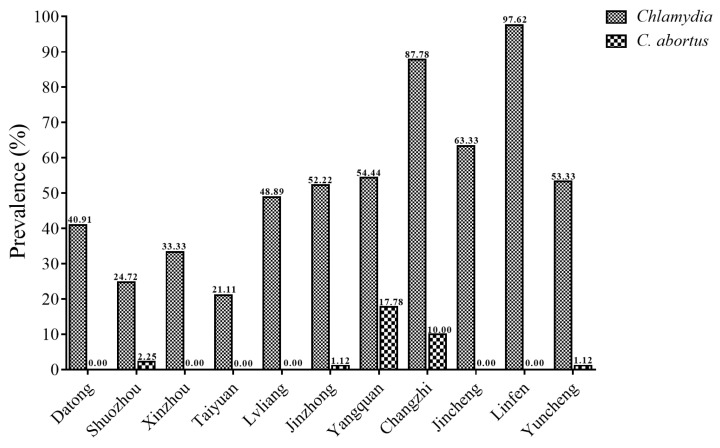
The seroprevalence of *Chlamydia* and *C. abortus* infection in cattle in 11 administrative cities in Shanxi Province, north China.

Among 981 cattle serum samples, the total seroprevalence of *Chlamydia* in Shanxi Province was 52.29% (513/981, 95% CI 49.17–55.42), with IHA titers of 1:16 in 408 cattle sera and 1:64 in 105 cattle sera (Table 2 and Table 3). The seroprevalence of *Chlamydia* varied greatly from 21.11% in Taiyuan to 97.62% in Linfen (Table 2 and Figure 2). In terms of location, the highest seroprevalence of *Chlamydia* infection in cattle was found in southern Shanxi Province (75.14%, 95% CI 70.64–79.64), followed by central Shanxi Province (44.17%, 95% CI 39.04–49.30) and northern Shanxi Province (32.96%, 95% CI 27.32–38.60). The seroprevalences of *Chlamydia* in cattle in the three management patterns (Household animal farm, Animal farming cooperative, and Large-scale animal farming company) were 49.03% (95% CI 44.92–53.14), 47.00% (95% CI 37.22–56.78), and 59.87% (95% CI 54.45–65.29), respectively. The seroprevalences of *Chlamydia* in cattle in different locations and management patterns were statistically significantly different (*p* < 0.01) (Table 3).

In this study, the total seroprevalence of *C. abortus* was 2.96% (29/981, 95% CI 1.90–4.02) in cattle in Shanxi Province (Table 4). Among the different cities, Yangquan had the highest seroprevalence of *C. abortus* (17.78%), followed by Changzhi (10.00%) and other cities (lower or 0) (Table 2 and Figure 2). There was a statistically significant difference in the seroprevalence of *C. abortus* between northern, central and southern Shanxi Province (*p* < 0.05). The highest seroprevalence of *C. abortus* in cattle was detected in the large-scale animal farming companies (3.82%, 12/314, 95% CI 1.70–5.94), followed by 3.00% (17/567, 95% CI 1.59–4.40) in the household animal farms and 0.00% in animal farming cooperatives. However, the seroprevalence of *C. abortus* in cattle in this study did not differ significantly between different management patterns (*p* > 0.05) (Table 4).

## 4. Discussion

The overall seroprevalence of *Chlamydia* infection in cattle in Shanxi Province was 52.29%, which was higher than that in Guangzhou (7.25%) [14]. The prevalence of *Chlamydia* has been reported in many other countries, such as Great Britain (31%) [15], Germany (50.8%) [16] and Poland (36.9%) [17]. The seroprevalence of *C. abortus* in cattle in Shanxi Province (2.96%) was lower than that in Tibet Autonomous Region (23.81%) [18], Gansu Province (16.22%) [19], Hebei Province (11.80%), and Shandong Province (12.67%) in China [9]. Similarly, *C. abortus* is widely found in cattle around the world, such as Switzerland (14.6%) [20] and Argentina (4.78%) [21]. The different seroprevalence of *Chlamydia* and *C. abortus* both within China and abroad may be due to the differences in national plans to control and eradicate the pathogens, detection methods, susceptibility of different breeds to pathogens, breeding environment, geographic location, and climate in the examined locations.

Given the significant differences in temperature and humidity between the north and south of Shanxi Province, the location was used as a factor to explore *Chlamydia* infection in cattle in Shanxi Province. As expected, the results showed that location was a significant risk factor for *Chlamydia* infection in cattle in Shanxi Province (*p* < 0.01) (Table 3). Compared to cattle in northern Shanxi Province, cattle in central and southern Shanxi Province had 1.61 times (95% CI 1.16–2.24) and 6.15 times (95% CI 4.33–8.73) higher risk of acquiring the infection, respectively. The high seroprevalence of *Chlamydia* in cattle in southern Shanxi Province may be due to the higher temperature and humidity in this area, and previous studies showed that animals have a higher seroprevalence of *Chlamydia* in summer or during the wet season [22,23]. It is worth noting that Datong (the northernmost city in Shanxi Province) had lower temperature and humidity than other northern cities, but the seroprevalence of *Chlamydia* showed the opposite trend. The main cause for this phenomenon may be that Datong is located at the throat of the Shanxi–Hebei–Mongolian Great Wall Golden Triangle cooperation zone, and there may be a certain risk of cross infection among cattle. Within southern Shanxi Province, *Chlamydia* seroprevalence in cattle in Changzhi and Linfen was higher than in surrounding cities. This may be due to Changzhi being located in the rainy belt of the Taihang Mountains of southeast Shanxi Province, which may have facilitated a high prevalence of *Chlamydia*. Disruption of the natural environment increases animal exposure to vectors and hosts of unknown pathogenic microorganisms [24]. For Linfen, its air pollution ranks first in Shanxi Province, which may increase the possibility of *Chlamydia* transmission. 

In addition to the immutable factor of location, an essential factor, management pattern, was investigated for its influence on *Chlamydia* infection in cattle in Shanxi Province. The seroprevalences of *Chlamydia* in cattle in different management patterns were significantly different (*p* < 0.01), ranging from 47.00% (95% CI 37.22–56.78) to 59.87% (95% CI 54.45–65.29) (Table 3). This indicated that management pattern was a risk factor for *Chlamydia* infection in cattle in Shanxi Province. Cattle in large-scale animal farming companies had a risk 1.68 times (95% CI 1.07–2.65) higher of acquiring *Chlamydia* infection than those in animal farming cooperatives. This may be related to breeding density, breeding environment and grazing conditions. Different breeding environment and grazing conditions may lead to differences in the proportion and types of microorganisms in the digestive system of cattle, leading to low immunity and increasing the incidence of chlamydiosis [25]. In addition, the suitability of breeding density also influences the spread of *Chlamydia* [26]. Furthermore, the vast majority of cattle farms in Shanxi Province use artificial insemination to breed calves to create greater economic value. Studies have demonstrated the presence of *Chlamydia* in artificial insemination bull semen, suggesting the possibility of sexual transmission [16]. This may also be one of the reasons why *Chlamydia* infection persists in cattle in Shanxi Province. For cattle herds, vaccination, such as inactivated vaccines and attenuated vaccines, is the most effective preventive measure, but poor immunogenicity and easy recurrence of virulence are its defects [27,28]. For large-scale *Chlamydia* infection in cattle, antibiotics are a good choice in terms of use and efficacy; however, antibiotics fail to be used frequently due to bacterial resistance and drug residues [10]. Cattle farms should carry out regular inspection of bulls and take timely measures to prevent the spread of infection once *Chlamydia*-positive cattle are found. In addition, regular disinfection and ventilation are also effective means to reduce the incidence of *Chlamydia* infection.

In view of the potential harm that *C. abortus* can cause to humans along with animals, this study also investigated *C. abortus* infection in cattle in Shanxi Province by two factors (location and management pattern). The seroprevalence of *C. abortus* in cattle in different locations was significantly different (*p* < 0.05). Compared with cattle in northern Shanxi Province (0.75%, 95% CI 0–1.78), cattle in central (4.72%, 95% CI 2.53–6.91) and southern Shanxi Province (2.82%, 95% CI 1.10–4.55) had more than six times (OR = 6.57, 95% CI 1.50–28.67) and nearly four times (OR = 3.85, 95% CI 0.84–17.73) higher risk of acquiring *C. abortus* infection, respectively (Table 4). These results were consistent with previous reports that location is a risk factor for *C. abortus* infection, such as in goats [29]. Nevertheless, management pattern was not a risk factor for the prevalence of *C. abortus* in cattle in Shanxi Province (*p* > 0.05) (Table 4). Four *Chlamydia* species were identified in cattle, namely *C. psittaci*, *C. pecorum*, *C. abortus* and *C. suis* [30]. Chlamydiosis in cattle is associated with a variety of diseases ranging from acute to chronic conditions [31]. *C. psittaci* causes acute bronchopneumonia after infecting calves and may result in long-term health impairments [32]. Most of the cattle infected with *C. pecorum* presented the symptoms of late-pregnancy abortion, calf meningoencephalitis and vasculitis [5]. Although *C. suis* has been found in cattle, no clinical symptoms were described [33]. The reproductive system of cattle infected with *C. abortus* is affected, with a variety of reproductive problems and miscarriage [34]. Therefore, although *C. abortus* was not the dominant species in cattle in Shanxi Province, attention still needs to be paid to its pathogenic characteristics and transmission route. 

Taken together, *Chlamydia* had a high seroprevalence (52.29%) in cattle in Shanxi Province, but *C. abortus* was not the dominant species. In contrast, the prevalence of species within the genus *Chlamydia* in China pointed to the most adapted species in cattle herds—*C. pecorum* or the dominant species in the detection of *Chlamydia* in cattle blood-*C. psittaci*, but this conjecture needs to be confirmed in cattle in Shanxi Province [33]. The temperature and humidity in southern Shanxi Province may be more suitable for the survival and spread of *Chlamydia* than in northern and central Shanxi Province. Eliminating the source of infection (e.g., timely elimination of *Chlamydia*-positive cattle, introduction of breeding bulls from epidemy-free areas) and cutting off routes of transmission (e.g., frequent ventilation to reduce aerosol concentrations) will effectively limit transmission by reducing the risk of exposure to *Chlamydia* and *C. abortus*.

## 5. Conclusions

The present study reveals the seroprevalence and risk factors of *Chlamydia* and *C. abortus* in cattle in Shanxi Province. The overall seroprevalence of *Chlamydia* and *C. abortus* was 52.29% and 2.96%, respectively. *Chlamydia* showed a high prevalence in cattle in Shanxi Province, especially in southern Shanxi Province. Location was identified as a risk factor for *Chlamydia* and *C. abortus* infection in cattle, whereas management pattern was identified as a risk factor for *Chlamydia* infection in cattle. This study provided baseline data for the prevention and control of *Chlamydia* infection in cattle in Shanxi Province.

## Figures and Tables

**Table 1:** Population and employment statistics of Shanxi Province.

Year	Resident Population	Rural	Primary Industry
2020	34.91	13.08	4.24
2019	34.97	13.53	4.62
2018	35.02	14.06	4.78
2017	35.10	14.54	5.00
2016	35.14	15.02	5.22
2015	35.19	15.53	5.50
2014	35.28	16.12	5.95
2013	35.35	16.66	5.92
2012	35.48	17.27	6.09
2011	35.62	17.88	6.50

Unit: million.

**Table 2:** Seroprevalence of *Chlamydia* and *C. abortus* in Cattle in 11 Cities of Shanxi Province.

Location	City	No. Examined	*Chlamydia* No. of Positive (%)	*C. abortus* No. of Positive (%)
Northern Shanxi	Datong	88	36 (40.91)	0 (0.00)
	Shuozhou	89	22 (24.72)	2 (2.25)
	Xinzhou	90	30 (33.33)	0 (0.00)
Central Shanxi	Taiyuan	90	19 (21.11)	0 (0.00)
	Lvliang	90	44 (48.89)	0 (0.00)
	Jinzhong	90	47 (52.22)	1 (1.12)
	Yangquan	90	49 (54.44)	16 (17.78)
Southern Shanxi	Changzhi	90	79 (87.78)	9 (10.00)
	Jincheng	90	57 (63.33)	0 (0.00)
	Linfen	84	82 (97.62)	0 (0.00)
	Yuncheng	90	48 (53.33)	1 (1.12)
Total		981	513 (52.29)	29 (2.96)

**Table 3:** Analysis of the related variables of *Chlamydia* infection in cattle in Shanxi Province.

Variable	Categories	No. Examined	No. Positive	Antibody Titers		Prevalence % (95% CI)	*p*-Value	OR (95% CI)
				1:16	1:64			
Location	Northern Shanxi	267	88	83	5	32.96 (27.32–38.60)	<0.01	Reference
	Central Shanxi	360	159	123	36	44.17 (39.04–49.30)		1.61 (1.16–2.24)
	Southern Shanxi	354	266	202	64	75.14 (70.64–79.64)		6.15 (4.33–8.73)
Management pattern	Household animal farm	567	278	237	41	49.03 (44.92–53.14)	<0.01	1.09 (0.71–1.66)
	Animal farming cooperative	100	47	43	4	47.00 (37.22–56.78)		Reference
	Large-scale animal farming company	314	188	128	60	59.87 (54.45–65.29)		1.68 (1.07–2.65)
Total		981	513	408	105	52.29 (49.17–55.42)		

**Table 4:** Analysis of the related variables of *C. abortus* infection in cattle in Shanxi Province.

Variable	Categories	No. Examined	No. Positive	Prevalence % (95% CI)	*p*-Value	OR (95% CI)
Location	Northern Shanxi	267	2	0.75 (0–1.78)	<0.05	Reference
	Central Shanxi	360	17	4.72 (2.53–6.91)		6.57 (1.50–28.67)
	Southern Shanxi	354	10	2.82 (1.10–4.55)		3.85 (0.84–17.73)
Management pattern	Household animal farm	567	17	3.00 (1.59–4.40)	0.14	Reference
	Animal farming cooperative	100	0	0.00		
	Large-scale animal farming company	314	12	3.82 (1.70–5.94)		1.29 (0.61–2.73)
Total		981	29	2.96 (1.90–4.02)		

## Data Availability

All datasets generated for this study are included in the article.
